# The complete mitochondrial DNA sequence of *Pectenocypris* sp. (Actinopterygii: Cyprinidae) from Serkap River, Sumatra, Indonesia

**DOI:** 10.1080/23802359.2018.1424585

**Published:** 2018-01-11

**Authors:** Dwi Atminarso, Arif Wibowo, Wahyu Endra Kusuma, Eko Prianto, Harald Ahnelt, Anti Vasemägi, Yoshinori Kumazawa

**Affiliations:** a Research Institute for Inland Fisheries, Agency for Research and Human Resource Development, Ministry of Marine Affairs and Fisheries, Palembang, Indonesia;; b Inland Fishery Resources Development and Management Department, Southeast Asian Fisheries Development Center, Palembang, Indonesia;; c Department of Aquaculture, Faculty of Fisheries and Marine Science, University of Brawijaya, Malang, Indonesia;; d Research Center for Fisheries, Agency for Research and Human Resource Development, Ministry of Marine Affairs and Fisheries, Jakarta, Indonesia;; e Department of Theoretical Biology, University of Vienna, Vienna, Austria;; f Department of Biology, Division of Genetics and Physiology, University of Turku, Turku, Finland;; g Institute of Veterinary Medicine and Animal Sciences, Estonian University of Life Sciences, Tartu, Estonia;; h Department of Information and Basic Science and Research Center for Biological Diversity, Graduate School of Natural Sciences, Nagoya City University, Nagoya, Japan

**Keywords:** Phylogenetic tree, mitochondrial genome, freshwater fish, Cyprinidae, *Pectenocypris*

## Abstract

The whole mitochondrial genome of a small cyprinid freshwater fish *Pectenocypris* sp. collected from Serkap River, Central Sumatra, Indonesia was sequenced. This mitochondrial genome consisted of 16,589 bp and included 37 genes in the same order as in many other vertebrates including the human. Phylogenetic analysis suggested that this taxon clusters with *Boraras maculatus* among several *Rasbora* species.

*Pectenocypris* (Actinopterygii: Cyprinidae) consists of four known freshwater fish species which are distributed in Sumatra and Borneo Islands: *P. balaena*, *P. korthausae*, *P. micromysticetus* and *P. nigra*. They are phytoplankton filter feeders (Rainboth 1991) but also feed on zooplankton (Kottelat 1982; Roberts 1989). All the species are small in size (<45 mm in standard length) and typically occur in acidic freshwaters. They have slender body, numerous and extraordinary extended gill rakers and a unique shaped lower pharyngeal jaw (Kottelat 1982; Roberts 1989; Tan and Kottelat 2009; Wibowo et al. 2016).

We conducted field survey at freshwaters in Central Sumatra and found possibly new species assignable to *Pectenocypris*. An adult individual of the undescribed *Pectenocypris* species with a standard length 31.1 mm was collected in peat waters of the Serkap River system in the Riau Province near Pelalawan, Central Sumatra (geographic coordinate: 00°34'42”N, 102°39'17”E) in 2013. The whole body specimen was deposited to the Museum Zoologicum Bogoriense (MZB), Bogor under the catalogue number MZB 22148. A small portion of muscle tissue was excised under the dorsal fin and preserved in ethanol for subsequent DNA extraction by Genomic DNA Mini Kit (Geneaid/ New Taipei City). Mitochondrial DNA was purified by digesting the linear nuclear DNA with the exonuclease V under the Mseek protocol (Jayaprakash et al. 2015) and then sequenced using the NEXTflex^TM^ Rapid DNA-Seq kit (Bioo Scientific/ Texas) with the Illumina NextSeq platform. This resulted in a single, circular DNA sequence which was then blasted against the Mitofish database (Iwasaki et al. 2013) for the confirmation of a fish mitochondrial DNA.

The complete mitochondrial DNA sequence of *Pectenocypris* sp. thus determined (16,589 bp; INSD database accession number LC337232) consisted of 37 genes for 13 protein subunits, 22 tRNAs, and two rRNAs together with a major non-coding region in the same order as in human (Anderson et al. 1981) and many other vertebrates. All protein genes had an ATG start codon except for the cytochrome oxidase subunit 1, and NADH dehydrogenase subunits 4L and 5 genes, which started with GTG, GTG and ATA, respectively. Seven protein genes were terminated with the TAA stop codon and the remaining six genes required polyadenylation for the establishment of stop codons in mRNAs. All tRNA genes can be folded into the standard cloverleaf secondary structures (Kumazawa and Nishida 1993).

This is the first complete mitochondrial genome sequence from genus *Pectenocypris*. The phylogenetic tree (Figure 1) suggested that *Rasbora* is not monophyletic in relation to genera *Trigonostigma, Pectenocypris*, and *Boraras*, which is in agreement with earlier molecular studies (Rüber et al. 2007; Britz et al. 2009; Tang et al. 2010). It was also suggested that contrary to the earlier work (e.g. Tang et al. 2010) *Pectenocypris* sp. is more closely related to *Boraras maculatus* (dwarf rasbora) than to other rasborine cyprinids examined although the bootstrap probability for this relationship was not very high (67%; Figure 1).

**Figure 1.**
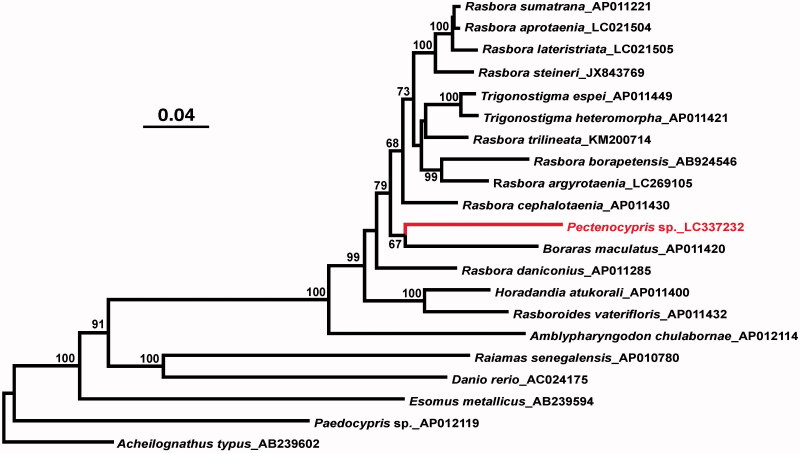
A maximum likelihood tree illustrating the phylogenetic position of *Pectenocypris* sp. among other rasborine cyprinids. The maximum likelihood analysis was conducted using concatenated amino acid sequences of 13 mitochondrial protein genes (3,813 sites) and Garli v2.0 (Zwickl 2017) under the mtREV + IG substitution model. Numbers at each node are bootstrap probabilities by 500 replications shown only when they are 50% or larger. INSD accession numbers of mitogenomic sequences for each taxon are shown along with the taxon name.
